# Corifollitropin alfa for poor responders patients, a prospective randomized study

**DOI:** 10.1186/s12958-020-00628-6

**Published:** 2020-07-09

**Authors:** F. M. Fusi, L. Zanga, M. Arnoldi, S. Melis, M. Cappato, I. Candeloro, A. Di Pasqua

**Affiliations:** Division of Reproductive Endocrinology, ASST Papa Giovanni XXIII, Piazza OMS 1, 24127 Bergamo, Italy

**Keywords:** Corifollitropin alfa, Agonist, Antagonist, Poor responders

## Abstract

**Background:**

Poor ovarian response remains one of the biggest challenges for reproductive endocrinologists. The introduction of corifollitropin alpha (CFA) offered an alternative option to other gonadotropins for its longer half-life, its more rapid achievement of the threshold and higher FSH levels. We compared two different protocols with CFA, a long agonist and a short antagonist, and a no-CFA protocol.

**Methods:**

Patients enrolled fulfilled at least two of the followings: AFC < 5, AMH < 1,1 ng/ml, less than three oocytes in a previous cycle, age > 40 years. Ovarian stimulation with an antagonist protocol was performed either with 300 UI rFSH and 150 UI rLH or 300UI HMG. In the long agonist group, after pituitary suppression with triptorelin, CFA was given the 1-2th day of cycle and 300 UI rFSH and 150 UI rLH the 5th day. In the short antagonist group CFA was given the 1-2th day of cycle and 300 UI rFSH and 150 UI rLH the 5th day. The primary objective was the effect on the number of oocytes and MII oocytes. Secondary objective were pregnancy rates, ongoing pregnancies and ongoing pregnancies per intention to treat.

**Results:**

The use of CFA resulted in a shorter lenght of stimulation and a lower number of suspended treatments. Both the CFA protocols were significantly different from the no-CFA group in the number of retrieved oocytes (*p* < 0,05), with a non-significant difference in favour of the long agonist protocol. Both CFA groups yielded higher pregnancy rates, especially the long protocol, due to the higher number of oocytes retrieved (*p* < 0,05), as implantation rates did not differ. The cumulative pregnancy rate was also different, due to the higher number of cryopreserved blastocysts (*p* < 0,02).

**Conclusions:**

The long agonist protocol with the addition of rFSH and rLH showed the best results in all the parameters. A short antagonist protocol with CFA was less effective, but not significantly, although provided better results compared to the no-CFA group. We suggest that a long agonist protocol with CFA and recombinant gonadotropins might be a valuable option for poor responders.

**Trial registration:**

The study was approved by the local Ethics Committee (EudraCT2015–002817-31).

## Background

Poor ovarian response remains one of the biggest causes of poor outcome in women undergoing ovarian stimulation, with a prevalence ranging from 9 to 24% [1]. Although in the literature a wide range of protocols using different doses and types of gonadotrophins to manage this particular cohort of patients have been proposed, to date the most efficient treatment for such a kind of patients is still unknown. Hence, the search for the best ovarian stimulation protocol for patients identified as ‘poor responders’ still continues [2–4]*.*

At first, the definition of poor responder has been a confounding matter, in that different criteria were used by reserchers. In any case, all the studies showed how pregnancy rates in poor ovarian responders remained substantially low [5]. In 2001 the definition of poor responders has been uniformed with the introduction of Bologna criteria [6], but still all the studies in patients fulfilling the Bologna criteria resulted in very low pregnancy rates, irrespective of patients’ age and type of ovarian stimulation protocol [4, 7].

One of the few stimulation protocols demonstrating promising pregnancy rates in these women is the administration of corifollitropin alfa (CFA) followed by highly purified human menopausal gonadotropin (hpHMG) in a gonadotropin-releasing hormone (GnRH) antagonist protocol [8].

Corifollitropin alfa is a recombinant dimeric glycoprotein, obtained fusing recombinant FSH with the carboxyterminal peptide of beta subunit of hCG. This fusion determines a half-life longer than FSH, a more rapid achievement of the threshold (24 h vs 48 h), and a higher level of FSH [9].

Recently, CFA has also been used with GnRH agonists, either in long protocol or with flare up protocol [10–12]. The rationale of the use of CFA in poor responders is related to its more precocious achievement of the threshold and to its higher level of FSH, that might allow the recruitment of a higher percentage of the few available follicles in these patients.

The present study was initiated with the aim of investigating whether the use of CFA, in association with recombinant FSH and LH, could be a useful tool for ovulation induction in poor responder patients, in a prospective randomized trial. In addition, the study evaluated the differences of the use of CFA in a long agonist, a short antagonist, both compared to cycles in which CFA was not utilized.

The study is a prospectic randomized, IRB approved study.

## Methods

### Study design

The study was a prospective, randomized, open study, performed from March 2016 to June 2019. For randomization, the criterion of allocation to each arm of the treatment was a computer-generated randomization sheet of the patients fulfilling the inclusion criteria. Patients were recruited in a ratio 1: 1 respectively for group A (controls without the use of CFA), B (long agonist group with the use of CFA) and C (Antagonist group with the use of CFA).

### Inclusion criteria

Patients who fulfilled at least two of the following criteria were admitted:

- Antral follicle count (AFC) < 5
- Antimullerian hormone (AMH) < 1,1 ng/ml
- Less than three oocytes obtained in the previous cycle
- Age > 40 years

### Exclusion criteria

- FSH > 20
- IV stage Endometriosis
- Severe male factor
- Body mass index (BMI) < 18 or > 30

#### Sample size

A total of 409 patients were enrolled in this study, and after randomization 136 were enclosed in group A, 137 in group B and 136 in group C. All the cases were with fresh embryos, and no PGT was performed. Most of the cases were treated with ICSI, a minority with IVF. The cases with IVF were 16 in group A, 20 in group B and 15 in group C. The embryo transfer was performed fresh in all cases.

### Ovulation induction

Patients were divided into three groups:

- Antagonist group without the use of CFA (Control)
- Long agonist with one dose of CFA
- Antagonist group with one dose of CFA

Group A):

- Administration of recombinant follicle stimulating hormone (rFSH) 300 UI and recombinant luteinizing hormone (rLH) 150 UI or human menopausal gonadotropin (HMG) 300 UI from the 3^th^day of the cycle
- Administration of a GnRH antagonist when the leading follicle reached 13 mm
- Final trigger using 10,000 UI of human chorionic gonadotropin (hCG)

Group B):

- Administration of triptorelin 0,1 from the 19th day of previous cycle to the day of final triggering
- Injection of corifollitropin alfa 100 or 150 based upon weight the 1st or 2nd day of the cycle
- Administration of rFSH 300 UI and rLH 150 UI from the 5th day after CFA injection
- Final trigger using 10,000 UI of hCG

Group C):

- Injection of corifollitropin alfa 100 or 150 based upon weight the 1st or 2nd day of the cycle
- Administration of rFSH 300 UI and rLH 150 UI from the 5th day after CFA injection.
- Administration of a GnRH antagonist when the leading follicle reached 13 mm
- Final trigger using 10,000 UI of hCG

In this study no blind steps were performed.

### Objectives

The primary objective of this study was the evaluation of the effect of CFA addiction on the number of total oocytes and MII oocytes retrieved in poor responder patients undergoing in vitro fertilization. Secondary outcomes were pregnancy rate, ongoing pregnancy rate and ongoing pregnancies per Intention to treat.

### Statistical analysis

Descriptive statistics were obtained for all the parameters. Mean and standard deviation were used for all quantitative parameters. Continuous variables were compared with the use of independent *t*-test. Differences between percentages were studied using Chi-square test generalized to the comparison of several proportions. The level of significance was set at *p* < 0.05.

The study was approved by the Local Ethics Committee (EudraCT 2015–002817-31, Deliberation 1400/2015), and Informed consent was obtained from all enrolled patients.

## Results

A comparison was done between the use of corifollitropin plus rFSH/rLH both using a long agonist or an antagonist protocol, and the use of an antagonist protocol without CFA with a maximal dose of HMG or rFSH/rLH (300 UI of FSH activity and 150UI of LH). The maximal dose was chosen due to the expected poor response or on the basis of previous results.

No differences were observed after randomization in the mean age of patients. The use of CFA resulted in a shorter length of stimulation in both CFA groups compared to the control (*p* < 0,05), with a not significant difference between the two CFA groups, although the antagonist CFA was the group with less days of treatment and less controls (Table 1). The number of suspended treatments for lack of response was significantly lower when using CFA for both protocols (*p* < 0,01 compared to controls). The group with the long agonist protocol and CFA had the higher values of Estradiol at triggering.

**Table 1:** Patients characteristics

	Control	Long agonist CFA	Antagonist CFA	
N patients	136	137	136	
Mean age	37,7 + 2,1	38,2 + 1,8	38 + 1,9	NS
Mean AFC	3,1 + 1,2	2,8 + 0,9	3,1 + 1,1	NS
Mean AMH (ng/ml)	0,7 + 0,3	0,6 + 0,2	0,6 + 0,3	NS
Days of stimulation	13,2 + 3,1	10,2 + 2,4	9,3 + 2,1	*p* < 0,05
Suspended treatments	16 (11,7%)	6 (4,3%)	8 (5,9%)	p < 0,05
N of controls	5,1 + 1,6	3,8 + 1,6	3,4 + 1,2	*p* < 0,05
E2 at trigger	846 + 283	1025 + 227	985 + 187	*p* < 0,05

When analyzing results, both CFA protocols were significantly different from the control group in the number of retrieved oocytes, number of MII oocytes and number of embryos (*p* < 0,05), with a non-significant difference in favour of the long protocol (Table 2). Patients who received a long CFA protocol showed a significant difference compared to control group in term of pregnancies, ongoing pregnancies per transfer and ongoing pregnancies per intention to treat (*p* < 0,05). The group that used antagonists and CFA showed a positive trend compared to control, and a negative trend compared to the long agonist CFA, but not significant (p < 0,5). The higher number of pregnancies was not due to any effect on the quality of embryos, but to the higher number of oocyte and consequently embryos available for the transfer, as the implantation rate was similar in the three groups (respectively 9,5; 10,3 and 10,2).

**Table 2:** Results of the study

	Control	Long agonist CFA	Antagonist CFA
Retrieved oocytes	3,6 + 2,2	5,5 + 2,3*	4,8 + 2,1*
N of MII oocytes	2,1 + 1,6	3,7 + 1,7*	3,4 + 1,5*
N of Embryos	1,8 + 0,8	2,9 + 0,5*	2,5 + 0,6*
N of no transfer	13/136 (9,5%)	6/137 (4,3%)**	9/136 (6,6%)*
Pregnancies/transfer	18/123 (14,6%)	26/131 (19,8%)*	21/127 (16,5%)
Ongoing pregnancies/transfer	13/123 (10,5%)	21/131 (16,1%)*	17/127 (13,4%)
Ongoing pregnancies/ITT	13/136 (9,5%)	21/137 (15,3%)*	17/136 (12,5%)

*p < 0,05 compared to control

**p < 0,01 compared to control

Only six of the patients of control group had blastocysts to be vitrified (4,4%) with no cumulative pregnancies. In the long agonist group 24 patients had a cryopreserved blastocyst (17,5%), and in the antagonist CFA 15 patients (11,1%). The cumulative pregnancy rate in this two groups, given that not all the patients utilized yet frozen blastocysts, increased respectively to 27,4% and 21,2%, with an ongoing pregnancy rate of 24,3% and 19,2% (*p* < 0,01 compared to cumulative pregnancy rate in controls).

Compared to the previous cycles, in those patients who were not at the first treatment, all the groups had an improvement of the number of oocytes obtained, with a higher significance for the groups that used a long agonist protocol (Table 3). The group that did not use CFA showed a tendential improvement compared to the previous cycle but not significant, probably due to the use of higher doses of gonadotropins.

**Table 3:** Comparison with the results of previous cycles

	Previous cycles	Control	Long agonist CFA	Antagonist CFA
**Number of patients**		32	38	36
**Mean collected oocytes**	2,8 + 1,3	3,1 + 1,1°	5,21 + 1,6*	3,8 + 0,9**
**Mean MII oocytes**	2,1 + 0,8	2,2 + 1,1°	4,64 + 0,6*	2,7 + 1,1°
**Mean Embryos obtained**	1,3 + 0,4	1,5 + 0,3°	4,2 + 0,5*	2,3 + 0,6**

*= p < 0,01 compared to previous cycles

**=p < 0,05 compared to previous cycles

° = Not significant

## Discussion

The best treatment for poor responders has still to be defined. Several studies comparing the GnRH antagonist versus short GnRH agonist [13, 14] or versus long GnRH agonist protocols [15, 16] in poor responders showed different and unclear results. A randomized controlled trial comparing the GnRH antagonist, the long agonist,and the short agonist protocols in poor responders demonstrated that the GnRH antagonists and the long GnRH agonist were comparable in terms of number of oocytes retrieved, as opposed to the short GnRH agonist protocol that led to inferior results [3].

The introduction of corifollitropin alpha opened new perspectives for the treatment of poor responders, because it has been shown to reach a peak of circulating levels within 2 days, whereas daily FSH does it after 3–5 days, and an higher level over the threshold [17]. This is advantageous for CFA compared to rFSH in that follicular recruitment depends upon the starting dose and cannot be modified by increasing FSH dose after 5 days. The results of the use of CFA in poor responders were very controversial, probably due to the small numbers of enrolled patients, and the different criteria used to define a poor responder. A pilot study with the use of CFA in an antagonist protocol has shown that ongoing pregnancy rates are low in these women, similar to the treatment with a short agonist protocol [8]. In a multicenter study, Drakopulos showed that CFA and HMG did not give any advantage in young poor responders compared to rFSH [18]. On the contrary, CFA followed by hp-HMG in a GnRH antagonist protocol resulted in promising pregnancy rates in poor ovarian responders < 40 years of age in a study of Polyzos [19]. Another recent observational cohort study demonstrated that in women with a low AFC, the number of oocytes retrieved is comparable between CFA and daily rFSH [20].

In this study, we added recombinant FSH and LH, at the 2:1 ratio, to corifollitropin alfa. The beneficial effect of such a protocol can be linked to a combined effect of both corifollitropin alfa and recombinant gonadotropins. Corifollitropin alpha is able to reach the threshold rapidly, with a circulating level of FSH well above it, thus allowing a wider recruitment of follicles [8]. rFSH from the 5th day from CFA, the day when circulating level star to decrease, was given to maintain the FSH effect and rLH was added to complete maturation of oocytes, although its role is still controversial, and to improve endometrial receptivity [21, 22].

The studies performed with CFA in long agonists protocols [12, 17], showed that CFA can be safely used in normal and poor responders. The use of antagonist protocols or long agonist protocols in poor responders show comparable results, better than those observed with short agonist protocols [3]. Our study was designed to evaluate the effect of addition of CFA in a long agonist or short antagonist protocol on oocyte yield in poor responders, when recombinant gonadotropins were added from the fifth day, earlier than usual protocols. Our data suggest that the long agonist protocol with CFA and the addition of rFSH/rLH from the fifth day obtain the best results, both in the number of retrieved oocytes and in fertilization rate. It was observed a significant increase in pregnancy, ongoing pregnancy and cumulative pregnancy rates. This increase seems not to be due to an improvement of the oocyte quality, but simply to the higher number of oocytes obtained, but further studies are needed. The fact that the number of cycles suspended for lack of response was significantly lower compared to controls resulted in a significant difference in number of ongoing pregnancies per intention to treat. The antagonist protocol with CFA yielded inferior results, although better than cycles with antagonists without CFA.

## Conclusions

We suggest that CFA in a long agonist protocol can be an interesting approach for poor responders. The addition of recombinant gonadotropins to corifollitropin alfa is also a new approach for these patients, both in a long agonist and a short antagonist protocol. The strength of this study is related to the number of patients enrolled, all poor responders based on previous cycles or markers of ovarian reserve, and to the fact that it was a prospective, randomized study.

## Data Availability

All data generated or analysed during this study are included in this published article.
